# An Overview of REBA Method Applications in the World

**DOI:** 10.3390/ijerph17082635

**Published:** 2020-04-12

**Authors:** Manuel Hita-Gutiérrez, Marta Gómez-Galán, Manuel Díaz-Pérez, Ángel-Jesús Callejón-Ferre

**Affiliations:** 1 Department of Engineering, University of Almería, Research Center CIMEDES (CeiA3), 04120 Almería, Spain; manuelhitagutierrez@gmail.com (M.H.-G.); mgg492@ual.es (M.G.-G.); madiaz@ual.es (M.D.-P.); 2 Laboratory-Observatory Andalusian Working Conditions in the Agricultural Sector (LASA), 41092 Seville, Spain

**Keywords:** musculoskeletal disorders, safety and health, biomechanics, physical load

## Abstract

The objective of this work is to review literature, worldwide, in which the Rapid Entire Body Assessment (REBA) ergonomic assessment method was applied and count the number of times that REBA was applied together with other methods and subsequent incidence. The database used was the “Web of Science—Core Collection”. Only scientific articles and bibliographic reviews were included, analysing a total of 314 documents and selecting only 91. The use of the REBA method is indicated in terms of knowledge, country, year and journal sectors. It was most used in the knowledge areas of “Manufacturing” (24.18%), “Agriculture, forestry and fishing” (21.98%) and in “Other activities” (19.78%). One of the benefits of REBA is that it evaluates different body parts: upper limbs (arm, forearm and wrist), lower extremities, trunk and neck. It is a useful method to identify the forced postures adopted by workers to thus develop improvement measures if necessary. It is concluded that REBA method use has increased over the last decade, probably due to the digitization of knowledge. It is almost always applied in combination with other methods, and its use can be a positive indicator of company sustainability.

## 1. Introduction

### 1.1. Musculoskeletal Disorders

According to the International Ergonomics Association [1], “ergonomics (or human factors) is the scientific discipline concerned with the understanding of interactions among humans and other elements of a system, and the profession that applies theory, principles, data, and methods to design in order to optimize human well-being and overall system performance”.

The World Health Organization (WHO) states that Musculoskeletal Disorders (MSD) “range from those that arise suddenly and are short-lived, such as fractures, sprains and strains, to lifelong conditions associated with ongoing pain and disability”. These disorders occur in people of any age and in all parts of the world. This disease has important economic consequences and implies a decrease in job performance, in addition to affecting the health of people who suffer from them [2].

The National Institute for Occupational Safety and Health (NIOSH) [3] defines musculoskeletal disorders as “a set of injuries and symptoms affecting the osteomuscular system and associated structures, such as bones, muscles, joints, tendons, ligaments, nerves and the circulatory system”.

To combat MSDs, ergonomic assessment methods are used to identify and assess the risk factors present in the workplace, and then, based on the results obtained, to propose redesign options that reduce the risk to acceptable exposure levels for the worker [4].

Currently, given that applying ergonomic methods is sometimes tedious because of the number of aspects to consider, software exists that makes it much easier, in terms of time and efficiency, to obtain the final results [5].

### 1.2. Assessment Methods

**Figure 1:**
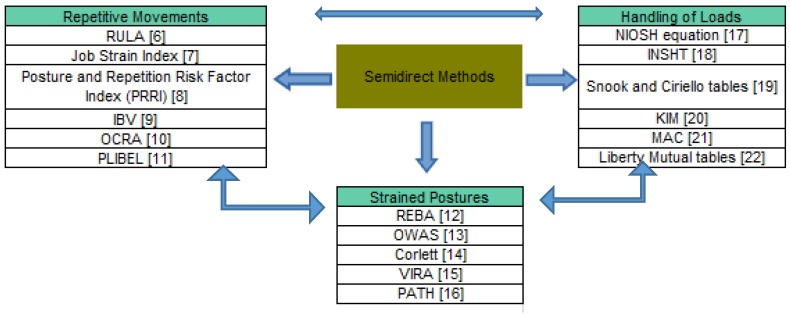
Semi-direct methods according to the cause of the musculoskeletal disorders and their combined use (adapted from Reference [5]). Rapid Upper Limb Assessment (RULA) [6]; Job Strain Index (JSI) [7]; Posture and Repetition Risk Factor Index (PRRI) [8]; Instituto de Biomecánica de Valencia (In Spanish; IBV) [9]; Occupational Repetitive Action (OCRA) [10]; Method for the identification of musculoskeletal stress factors which may have injurious effects (PLIBEL) [11]; Rapid Entire Body Assessment (REBA) [12]; Ovako Working Analysis System (OWAS) [13]; Corlett [14]; Video film technique for Registration and Analysis of working postures and movements (VIRA) [15]; Posture, Activity, Tools and Handling (PATH) [16]; National Institute of Occupational Safety and Health (NIOSH) [17]; Instituto Nacional de Seguridad e Higiene en el Trabajo (In Spanish; INSHT) [18]; Snook and Ciriello tables [19]; Key Indicator Method (KIM) [20]; Manual Handling Assessment Charts (MAC) [21]; Liberty Mutual tables [22].

The methods used for evaluating musculoskeletal disorders vary depending on the country, the companies carrying them out and the working environment, etc. For this reason, it is possible to classify them as direct, semi-direct or indirect methods [5]. Direct methods require electronic devices to be placed on the individual’s body, evaluating the worker in real time. Semi-direct methods (Figure 1) are based on images that are subsequently evaluated while indirect methods use questionnaires.

Semi-direct methods can be classified according to the cause of the MSD. In this study, the Rapid Entire Body Assessment (REBA) method is one of the methods used for assessing forced postures (Figure 1).

The application of methods such as REBA has evolved over time. It started with photographs, paper and pen. Over time, progress was made using video recordings and employing analysis of this through software. Currently, some equipment is used to measure angles and evaluate in real time [23,24].

### 1.3. The Rapid Entire Body Assessment Method (REBA), Justification and Objective

This method was developed by Sue Hignett and Lynn McAtamney at Nottingham Hospital (The United Kingdom) and published in 2000 [12]. It is the result of cooperative work carried out by teams of ergonomists, physiotherapists and nurses after identifying/analysing around 600 working postures. REBA allows one to jointly analyse the postures of the upper limbs (arm, forearm, wrist), trunk, neck and lower extremities. In addition, it discriminates the type of grip and muscle activity performed. It identifies five levels of risk, from negligible to very high [12].

The main advantages of the REBA method are [25]:

- The cost-effectiveness ratio is good.
- It is easy to apply. Pen and paper are enough for data collection; however, there are computer applications that speed up/facilitate its use.
- The most conflictive ergonomic aspects are identified from the individual score obtained after assessing each part of the body.

The main limitations are [25]:

- It only allows the analysis of individual postures. It is not possible to analyse a set or sequence of postures.
- Task evaluations will depend on the evaluator. Some of the positions adopted may or may not be examined.
- It only measures the effort intensity. The duration of exposure and the frequency of postures throughout the working day are not considered.

One of the requirements of the method is to have the consent of the worker to obtain the necessary information. The evaluators observe all the tasks to be analysed. Observation can be completed in three ways: direct observation, video recording or taking photographs. The aim is to collect data that allows the method to be used to obtain results.

On the other hand, the method presents some differences with respect to others. One of the main differences is that it considers the lower extremities of the worker [12]. These are not considered by other evaluation methods such as RULA [6]. There are no better or worse methods, but they are applied depending on the evaluators’ situations and resources [26].

It should be noted that once the method has been published, over the years, it is very important to know its worldwide application.

Examining the use of the REBA method, since its inception, would justify the impact of this method on society.

The main objective of this work is to carry out a bibliographic review of the REBA method [12] application in the fields of knowledge, countries, years and journals from the period May 2002 to July 2019. In addition, this study aims to count the number of times that REBA has been applied together with other methods and subsequent incidence. Finally, an objective is to demonstrate whether it has been applied in the health field and what happens with respect to the rest of the knowledge categories.

## 2. Materials and Methods

### 2.1. Searching for Information

In order to search for the information, electronic access to the library of the University of Almeria was used. Then the “Web of Science—Core Collection (WOS)” database was accessed. Its license is granted by Spanish Foundation for Science and Technology (FECYT).

It was assumed that all the researchers who applied REBA in any field of knowledge would have cited the article where this method was published. Therefore, the search process was performed to access all the studies that cited it. “Advanced Search” was used in WOS with the terms “so=applied ergonomics and ti=Rapid entire body assessment (REBA)”. In this way, a single result was obtained, which was the original article of REBA [12]. Accessing this result, the “Times Cited” section was consulted, which showed all the studies that had cited the REBA article [12]. There was a total of 442, in the period from 2002 to 10/07/2019.

Of the 442 citations, books, book chapters or other formats were discarded, finally obtaining 314 citations for articles and reviews. It is worth reiterating that only the WOS database was used.

Of these 314 results, the number of studies finally selected for this document was 91. These do not include the original article of the method. Some studies were discarded because they were repeated articles, sometimes as reprints or as conferences that were subsequently published in journals, so only the original article was considered. Others were discarded because, although they cited the paper on the REBA method, they did not apply it in the research. Only articles using REBA method were considered, individually or in combination with other methods.

### 2.2. Data Analysis

Variables, categories and their abbreviations are shown below (Table 1). In addition, all journals were considered.

The knowledge categories, countries and years were grouped to facilitate data management. Countries were grouped into continents. Not all countries on a continent are shown, only those where the REBA method was applied.

On the other hand, the 91 final studies were grouped according to field, year, country and journal. For the general grouping by sectors, an adapted classification was used [27]. Another classification was made within each sector, but in this case it was from the information obtained from studies analysed, for greater clarity and organization of this information.

XLSTAT2019 (Addinsoft, Paris, France) [28] software was used for the results analysis.

## 3. Results and Discussion

The frequencies of each variable category are shown in Table 2.

The area in which the method was applied most is the “Manufacturing (C)” followed by “Agriculture, forestry and fishing (A)” and “Other activities (OTH)”. Between the period 2014 and 2019 (Y5 and Y4), more than 70% of the REBA applications were published. Half of the studies with REBA were published in Asia (C2). The total number of journals is 91. The most striking observation is that, although the method was initially published in 2000, its application was not significant until about 15 years later, possibly because of the digitization of academic/scientific content and the massive user access via the internet [29]. This coincides with other applications concerning ergonomic assessment methods, namely the case of OWAS [5].

Risk assessment is mandatory in all companies. Each one chooses the evaluation method. REBA [12] allows for the identification of the musculoskeletal disorders suffered by workers in different fields, mainly forced postures.

### 3.1. Analysis by Field and Knowledge Categories

#### 3.1.1. Human Health and Social Work Activities

Table 3 shows studies related to human health and social work activities.

##### Hospitals

The REBA assessment method is sometimes combined with results support software, as was the case with Abdollahzade et al. [30] using SPSS in the study of 147 high-risk nurses in Tabriz, Iran. It is also common to create computer applications based on the REBA method. Janowitz et al. [35] created a hospital task-scoring algorithm while several authors [31] established a computer system based on information and communication technologies to support hospital processes.

In 2014, Kim and Roh [37] conducted a REBA method-based study on radiologists with more than five years of experience, demonstrating that MSDs occur mainly in the shoulder and lumbar regions, the same symptoms suffered by surgical nurses in a Portuguese hospital [42].

The combination of ergonomic methods developed by Ratzon et al. [41] in the study of 31 nurses, was not enough to determine the effect of poor postural habits on the job. Therefore, they recommend a longer period of study to see if the intervention might reduce MSDs.

##### Dentistry

Applying REBA to dental hygienists is a common practice in the study of plantar pressure [36] and in the taking of oral X-ray images [38]. On other occasions, it is combined with other methods, as was the case with Rafeemanesh et al. [40] in order to demonstrate that the neck area is the most vulnerable part for these professionals and to raise awareness of the importance of workplace design and rest periods during the activity as a basic prevention principle.

##### Gynaecology

Several authors [34] observed the need for engineering solutions that allow surgeons flexibility during their interaction with patients.

##### Otorhinolaryngology (ENT)

A study was carried out to evaluate the musculoskeletal disorders of ENT specialists in the surgical context. Their training and the ergonomic tools used were also analysed. The results show that only 24% of the workers were trained in ergonomics. It was also concluded that the workers adopted forced postures [44].

##### Others

According to Carneiro et al. [32], home-care nurses generally have a moderate postural assessment in their work activities.

When comparing different forms of tracheal intubation, several authors [33] used the REBA method to determine which technique, GlideScope or Macintosh, was less likely to cause musculoskeletal injuries during use, the former scoring the highest and therefore being the one chosen.

In the refitting of a vaccine production centre, Torres and Vina [43], in a study using REBA and NIOSH, established shelf redesigns and working method modifications as a measure to reduce the MSD risk level. On the other hand, Pascal and Naqvi [39] in a Canadian study outlined the need for retraining plans to help raise awareness of the risks posed by bad work activity practices.

#### 3.1.2. Agriculture, Forestry and Fishing

Table 4 shows the studies related to agriculture, forestry and fishing.

##### Forestry

In this sector, REBA began to develop in Canada and the US with the combination of five ergonomic assessment methods in sawmill facilities [54,55,56]. In addition, ErgoFellow software combined REBA and OWAS methods in the ergonomic assessment of forestry machinery use [57], concluding that chainsaw operator work is more demanding and riskier than that of wood collector operators over the course of the activity [52]. The same conclusions were drawn in the study on chainsaw use employing the OCRA, OWAS, RNLE equation and REBA methods [64]. In 2019, REBA and RULA were compared in wood chipping activity concluding that the latter offered greater risk prevention when applying the method [46]. Likewise, in 2017, several authors [60] used REBA to assess management worker tasks.

In forest nurseries, Unver-Okan et al. [63] combined several ergonomic methods to study working postures, such as seed sifting or machine sowing. They finally chose the RULA method for assessment because of its increased sensitivity in the final result.

##### Livestock

In the livestock field, Taghavi and Mokarami [62] used the REBA method to assess the postural burden related to feeding, milking and dung removal during milk production. In Brazil, numerous authors [50] did the same with regard to milking and livestock management activities, demonstrating a high level of risk over the course of the activity.

##### Agriculture

Das and Gangopadhyay [47] applied REBA in potato growers in order to assess musculoskeletal disorders. They showed that one of the most affected body areas was the lumbar region. The same was stated by Das et al. [49] who applied it in the same crop type, but in this case studying children. The method has also been used in the study of crops such as tomato [59,61], pepper [59], rice [58], oil palm [51] and apples [53], as well as in the collection of ornamental plants [45] and seeds [48].

#### 3.1.3. Manufacturing

Table 5 lists studies that used the REBA method in different areas of manufacturing.

##### Metallurgical Sector

The first analyses appeared in 2013 in Spain with REBA combined with RULA [71], and with BREBA [67] in 2015 in Turkey, the common goal being to eliminate economic risks and improve the production systems in factories. In the first case, this was achieved by means of simulations and, in the second, by means of photographs. Subsequently, several authors again combined methods (OWAS, RULA and REBA) [66,76] in iron and steel operations that involved milling, turning and drilling.

##### Textiles

Isler et al. [75] studied 65 operators from different departments (cutting, sewing, ironing, quality control, etc.) in eight companies using video cameras, resulting in a REBA score of more than 11 points; thus, they recommended immediate intervention. In Sri Lanka, 552 female foot-sewing-machine operators were tested, with the medium-high REBA intervention warning regarding problems in the knees, feet and thighs [79].

##### Technology

The application of REBA in manufacturing technology is defined by activities such as computer repair [77] or the manufacturing of electrical products [86]. In addition, it was combined with other methods such as OCRA to study the ergonomic assessment of electric motor assembly line operators [69]. Felekoglu and Tasan [70] have replaced traditional REBA assessment with Kinect sensors and electrogoniometers.

##### Production Lines

Many authors analysed production lines for automotive elements [84], plastics [83] or brick furnaces [81] using the REBA method. Conversely, Cornell University [72] developed a REBA-inspired musculoskeletal discomfort questionnaire, the AnyBody Modeling System (AMS) and electromyography measurements that identified the musculoskeletal disorders of employees on a harness assembly line.

At other times, the method was applied individually or in conjunction with other methods in packing factories dealing with varied orders [85], beverages [65] and even minerals [68], or in operations carried out by potters and sculptors [80] who suffer curvature in various parts of the body derived from forced postures.

In rubber production in Iran, Samanei et al. [82] combined the REBA and NMQ methods with a subsequent results analysis using SPSS software, concluding by identifying the need for immediate intervention in the lumbar region.

In sand-dredging operations in Udupi (India) [78], the REBA method, with a 12-point assessment, advised immediate intervention to reduce injuries, specifically in the lower back, as did the assessment by Hanson et al. [73] in manual collection operations. Furthermore, recent studies in the United Kingdom [74] gathered information on the effects of individual skills on job performance and safety in the workplace as well as human well-being, to design more inclusive work practices.

#### 3.1.4. Transportation and Storage

Table 6 shows the four publications that have applied REBA individually or collectively in the study of forced postures related to transportation and storage.

There were two publications combining RULA and REBA. In the first, Balaji and Alphin [88] took photographs of operators who handled industrial excavators, observing that 46% of workers were exposed to high levels of danger; this resulted in the tasks being optimized and redesigned. In the second, Bora et al. [89] evaluated posture parameters in industrial vehicles using CATIA software.

Ahmed et al. [87], using a combination of ergonomic methods, assessed bus drivers who transported people with reduced mobility, looking at three different wheeled mobility devices: manual, scooter and electric. The results determined a high level of risk during the WTORS (Wheelchair Clamping and Occupant Restriction System) procedure. In the railway sector, several authors [90] combined REBA and NMQ in the ergonomic study of 51 railway workers, determining that the shoulder was the most affected body area followed by the neck.

#### 3.1.5. Water Supply; Sewerage, Waste Management and Remediation Activities

Table 7 shows two studies related to waste management.

Cakit [91] combined the REBA and RULA methods in the study of waste collection movements, mainly in lifting and unloading tasks, considering it essential that these tasks be changed as soon as possible. Jozwiak et al. [92], used REBA, FirstBeat and stadiometry in the ergonomic study of urban solid waste collectors.

#### 3.1.6. Professional, Scientific and Technical Activities

Table 8 shows publications that focus on different professional, scientific and technical activities.

In professionals working with laboratory sample preparation [94], the REBA method assessed six subtasks for each of the six tasks analysed, concluding there was a medium-high risk level in at least one subtask for each task.

This method was also used, in combination with others, in sales assistants in Italy [93].

#### 3.1.7. Activities of Households as Employers; Undifferentiated Goods—and Services—Producing Activities of Households for Own Use

There are three publications related to activities of households in which the REBA method has been applied: Lim et al. [95], Lofqvist et al. [96] and Rui et al. [97], corresponding to vacuum cleaning work, basic household chores and tasks associated with drying clothes, respectively (Table 9).

#### 3.1.8. Education

In the field of education, only Hashim et al. [98] combined REBA and RULA to assess the different positions students take while they do their schoolwork, demonstrating that the majority need immediate intervention to prevent greater prejudicial effects (Table 10).

#### 3.1.9. Construction

Table 11 shows studies related to construction.

Several authors [102] created an ergonomic assessment tool using videos that allowed for postures to be assessed while working. Kim et al. [100], for their part, demonstrated how the manufacture of goods from prefabricated panels negatively influenced the spinal column of the workers.

In addition, other authors [101] made use of new 3D technologies to screen workers in different workplaces thus reducing the costs derived from MSDs and correcting bad habits. Conversely, in Western Bengal (India), Chatterjee and Sahu [99] combined different ergonomic assessment methods to demonstrate that a more conciliatory schedule and rest times, accompanied by technical modifications in the workplace and the use of redesigned equipment, reduced the risk of MSD.

#### 3.1.10. Other Activities

Table 12 includes studies that could not be classified in any of the above areas.

The REBA method has been translated into other languages such as Portuguese [110]. Its reliability was also evaluated in 2019 by Schwartz et al. [116]. Moreover, it has been compared to other methods [115], raising the possibility of creating a comprehensive method for all work tasks and all body parts. Diego-Mas et al. [107] produced a study that detected the anomalies of different ergonomic assessment methods.

In Jaipur, India, two studies by Mukhopadhyay analysed the ergonomic assessment of operators in three artisanal sectors [112] as well as in stone carving [113]. Other authors assessed African women as they carried out two tasks related to loading bricks on their heads [106] and the load variance during pregnancy [108]. Bicycle repair [111] and marine mollusc collection [109] were also assessed using the REBA method.

Furthermore, the use of ergonomic assessment methods is common in areas such as aircraft maintenance [103], electrical equipment manufacture [104], armament cleaning tasks [114], engine oil companies [118], various industrial sectors [105] and even in multitasking jobs [117].

Yuan [120] assessed 39 employees from nine different library divisions on two occasions by combining the REBA and RULA methods. The workers’ bad habits were improved once action guidelines were established.

Finally, REBA was also combined with other evaluation methods in the case of firefighters and medical emergency technicians [119].

### 3.2. Analysis by Country

**Figure 2:**
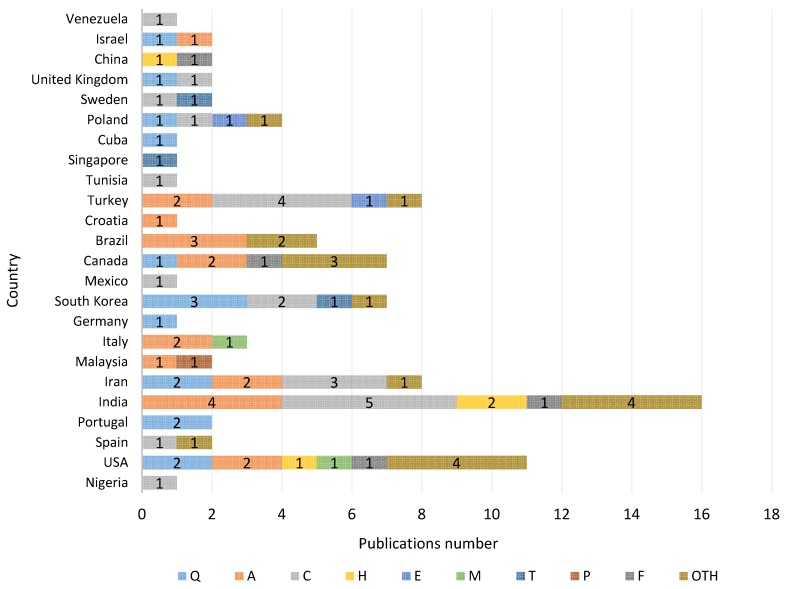
Publications by field and by country (see abbreviations Table 2).

The REBA method has been applied in 91 cases and in 24 different countries. The country where the highest number of studies has been carried out is India, with a total of 16; followed by the USA with 11; Iran and Turkey with eight; Canada and South Korea with seven; Brazil with five; Poland with four; Italy with three; Spain, Portugal, Malaysia, The United Kingdom, China, Sweden, and Israel with two, and Nigeria, Germany, Mexico, Croatia, Tunisia, Singapore, Cuba, and Venezuela with one publication each (Figure 2). One can observe that approximately 50% of the countries where REBA was used are in the process of developing.

In “manufacturing”, five REBA applications in India and four in Turkey stand out. In turn, in “agriculture, forestry and fishing”, four research studies in India and three in Brazil are highlighted. In “other activities”, four studies in the USA and India and three in Canada stand out.

The USA is one of the countries with the highest number of contributions over diverse fields: human health and social work activities (2), agriculture, forestry and fishing (2), transportation and storage (1), professional, scientific and technical activities (1), construction (1) and others activities (4).

If you look at the United Kingdom, the country where the REBA method arose, there were only two studies in which the method was applied. This fact is countered by the much higher use of other methods, such as OWAS [13], in its country of origin [5]. Perhaps this is because the REBA method is more recent (the year 2000) than OWAS (1977).

### 3.3. Analysis by Year

**Figure 3:**
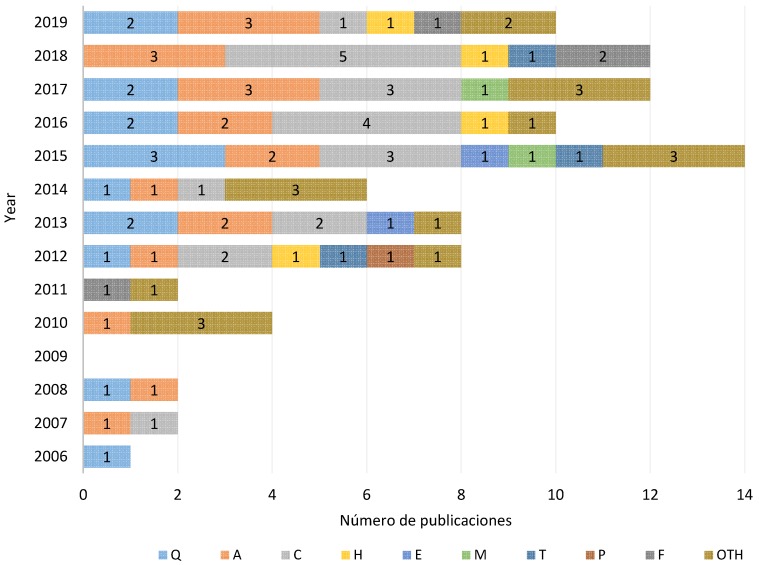
Publications by field and by year.

The year with the highest number of publications was 2015, when there were fourteen in seven different areas, followed by 2017 and 2018 with twelve publications and 2016 and 2019 with ten (Figure 3). The years when there were the lowest number of research studies were 2007, 2008 and 2011 with two publications and 2006 with only one. On the other hand, over the years spanning the bibliographic review, there is no indication of any REBA method being applied in 2009 (Figure 3).

Thanks to society’s awareness of the risks of MSD in the workplace, the publication of studies is growing exponentially over recent years, no doubt because of the developments in information and communication technologies, as mentioned before.

### 3.4. Analysis by Journal

The journals that most stand out are: “Work-A Journal of Prevention Assessment and Rehabilitation” with 18.68% of the publications and the “International Journal of Industrial Ergonomics” with 15.38% (Table 13). “Applied Ergonomics”, “Human Factors and Ergonomics in Manufacturing and Service Industries”, “Journal of the Faculty of Engineering and Architecture of Gazi University” and the “International Journal of Occupational Safety and Ergonomics” are next with 3.30% each (Table 13).

### 3.5. Combination with Other Methods

REBA is normally applied in conjunction with other assessment methods for musculoskeletal disorders. The REBA method [12] is mainly used to evaluate forced postures (Figure 1). However, it is sometimes combined with methods that study other risk factors. One of them, the RULA method, is also used to analyse repetitive movements (associated with REBA) [6]. Due to these considerations, some studies present a broader and more complete analysis [46,66].

In 47 of the studies reviewed, REBA is applied along with other methods [31,40,41,43,46,48,49,51,52,53,54,55,56,57,58,63,64,65,66,67,68,69,71,75,76,79,80,82,83,87,88,89,90,91,92,93,98,99,100,103,104,105,112,114,117,119,120]. These 47 results are included in the following categories: “human health and social work activities”, “agriculture, forestry and fishing”, “manufacturing”, “transportation and storage”, “water supply; sewerage, waste management and remediation activities”, “professional, scientific and technical activities”, “education”, “construction” and “other activities”. Of these, “agriculture, forestry and fishing” stands out with 13 studies, followed by “manufacturing” with 12. Thanks to the combined use of the methods, the upper limbs, trunk, neck and lower extremities can be evaluated with greater precision [12].

## 4. Conclusions

The REBA method is mainly used for the analysis of forced postures. It is not useful for the evaluation of repetitive movements.

Although this method was initially intended to be applied to the analysis of forced postures in personnel related to the human health and social work activities field, as well as various activities in the service sector, it can be applied to any sector or labour activity. In fact, it is observed that the application number is greater in other knowledge categories than in the original field.

In this literature review, the REBA method was mainly applied to three areas: “manufacturing”, “agriculture, forestry and fishing” and “other activities”.

It is often widely used in combination with other methods and has was greatly by the digitization of scientific content over the last decade.

In addition, unlike other methods which that are combined with REBA, this one focuses on the upper limbs (arm, forearm and wrist), lower extremities, trunk and neck.

In the Americas, its application is highly diversified over the different fields of knowledge. Conversely, in Asia, it is applied more specifically in two sectors: “manufacturing” and “agriculture, forestry and fishing”.

In countries immersed in the development process, it is not easily used since they do not have enough technology or information on the method. However, the fact that approximately 50% of the studies were carried out in developing countries may be an indicator of sustainable processes starting in companies there.

The journals that have published the most studies on applying the REBA method are “Work-A Journal of Prevention Assessment and Rehabilitation” and the “International Journal of Industrial Ergonomics”.

## Figures and Tables

**Table 1:** Variables, categories, and abbreviation.

Variable	Categories	Abbreviation
Sector	Agriculture, forestry and fishing	A
	Manufacturing	C
	Transportation and storage	H
	Water supply; sewerage, waste management and remediation activities	E
	Professional, scientific and technical activities	M
	Activities of households as employers; undifferentiated goods—and services—producing activities of households for own use	T
	Construction	F
	Education	P
	Human health and social work activities	Q
	Other activities	OTH
Year	Items between 2006 or before	Y1
	Items between 2007 and 2010	Y2
	Items between 2011 and 2013	Y3
	Items between 2014 and 2016	Y4
	Items between 2017 and 2019	Y5
Country	Europe: United Kingdom, Sweden, Poland, Croatia, Italy, Germany, Portugal and Spain	C1
	Asia: Israel, Iran, India, South Korea, China, Singapore, Malaysia and Turkey	C2
	America: USA, Mexico, Brazil, Canada, Venezuela and Cuba	C3
	Africa: Tunisia and Nigeria	C4
Journal	-	-

**Table 2:** Category frequencies.

Variable	Category	Frequency	%
Sector	A	20	21.98
	C	22 *	24.18
	E	2	2.20
	F	4	4.40
	H	4	4.40
	M	2	2.20
	OTH	18	19.78
	P	1	1.10
	Q	15	16.48
	T	3	3.30
Year	Y1	1	1.10
	Y2	8	8.79
	Y3	18	19.78
	Y4	30	32.97
	Y5	34 *	37.36
Country	C1	17	18.68
	C2	46 *	50.55
	C3	26	28.57
	C4	2	2.20
Journal	-	91	100

* Mode.

**Table 3:** Human health and social work activities.

Reference	Country	Year	Objective
[30]	Iran	2016	REBA with SPSS in the ergonomic assessment of operating room nurses.
[31]	Poland	2015	Combination of ergonomic assessment methods on nursing and surgery personnel.
[32]	Portugal	2015	REBA on home-care nurses.
[33]	Germany	2015	REBA in comparing GlideScope and Macintosh in the tracheal intubation process.
[34]	United Kingdom	2017	REBA in the gynaecological field.
[35]	USA	2006	REBA for creating a hospital task scoring algorithm
[36]	South Korea	2019	REBA on dental hygienists.
[37]	South Korea	2014	REBA on radiologists.
[38]	South Korea	2013	REBA on dental hygienists.
[39]	Canada	2008	Readapting plans to help overcome bad practice in work activities.
[40]	Iran	2013	REBA and NMQ (Standardised Nordic questionnaires for the analysis of musculoskeletal symptoms) on the ergonomic assessment of dentists.
[41]	Israel	2016	REBA, NMQ and Karasek on the ergonomic assessment of nurses in hospitals.
[42]	Portugal	2017	REBA on nurses.
[43]	Cuba	2012	REBA and NIOSH for the refitting of a vaccine production centre.
[44]	USA	2019	REBA on otolaryngology surgeons.

**Table 4:** Agriculture, forestry and fishing.

Reference	Country	Year	Objective
[45]	Brazil	2015	REBA in the collection of ornamental plants.
[46]	Italy	2019	RULA and REBA on wood chippers in the forestry sector.
[47]	India	2015	REBA on potato growers.
[48]	India	2012	NMQ and REBA on seed collectors.
[49]	India	2013	NMQ, REBA and OWAS in the ergonomic assessment of child potato growers.
[50]	Brazil	2018	REBA in livestock activities.
[51]	Malaysia	2016	NMQ and REBA in oil palm plantations.
[52]	Turkey	2019	OWAS and REBA in wood harvesting in the forestry sector.
[53]	Iran	2018	NMQ and REBA in apple harvesting.
[54]	Canada	2007	Five ergonomic assessment methods in sawmill installations in the forestry sector.
[55]	Canada	2008	Five ergonomic assessment methods in sawmill installations in the forestry sector.
[56]	USA	2010	Five ergonomic assessment methods in sawmill installations in the forestry sector.
[57]	Croatia	2019	ErgoFellow, REBA and OWAS in the ergonomic assessment of forestry machinery use.
[58]	India	2018	Four ergonomic assessment methods in rice cultivation by women.
[59]	Israel	2016	REBA on special greenhouse crops.
[60]	Brazil	2017	REBA in the ergonomic assessment of forestry machinery.
[61]	USA	2014	REBA on tomato cultivation.
[62]	Iran	2017	REBA on dairy production.
[63]	Turkey	2017	Various ergonomic methods on forest nursery workers.
[64]	Italy	2013	Four ergonomic methods in the ergonomic study of forestry machinery use.

**Table 5:** Manufacturing.

Reference	Country	Year	Objective
[65]	Nigeria	2016	REBA and NMQ on beverage bottlers.
[66]	Tunisia	2018	REBA and RULA in milling, turning and drilling operations.
[67]	Turkey	2015	REBA and BREBA in the metallurgical sector.
[68]	Iran	2016	REBA in mineral packers.
[69]	Venezuela	2012	REBA and OCRA in electric motor assembly.
[70]	Turkey	2017	Ergonomic comparison between the Kinect sensor and electrogoniometer to assess posture.
[71]	Spain	2013	REBA and RULA in the metallurgical sector
[72]	Turkey	2018	Creation of an ergonomic assessment questionnaire at Cornell University for the manufacture of harnesses.
[73]	Sweden	2018	REBA in manual collection operations.
[74]	United Kingdom	2016	Research on work practices.
[75]	Turkey	2018	Ergonomic methods in the textile industry.
[76]	South Korea	2007	OWAS, RULA and REBA in the metallurgical sector.
[77]	Mexico	2015	REBA in computer repair.
[78]	India	2017	REBA in sand dredging in Karnataka.
[79]	India	2019	Ergonomic methods in the Sri Lankan textile industry.
[80]	India	2013	RULA and REBA in operations carried out by potters and sculptors.
[81]	India	2018	REBA in brick kiln workers in Rajasthan.
[82]	Iran	2017	REBA and NMQ in the rubber industry.
[83]	India	2015	Ergonomic methods in the plastic furniture manufacturing industry.
[84]	South Korea	2016	REBA on automotive assembly lines.
[85]	Poland	2014	REBA in the packaging industry.
[86]	Iran	2012	REBA in an electrical products factory.

**Table 6:** Transportation and storage.

Reference	Country	Year	Objective
[87]	USA	2012	Ergonomic methods in the transport of people with reduced mobility.
[88]	India	2016	REBA and RULA in industrial vehicle operations.
[89]	China	2019	REBA and RULA on industrial vehicle drivers.
[90]	India	2018	REBA and NMQ in the railway sector.

**Table 7:** Water supply; sewerage, waste management and remediation activities.

Reference	Country	Year	Objective
[91]	Turkey	2015	REBA and RULA in waste collection tasks.
[92]	Poland	2013	REBA, Firstbeat and stadiometry in the ergonomic study of solid waste collectors.

**Table 8:** Professional, scientific and technical activities.

Reference	Country	Year	Objective
[93]	Italy	2017	Ergonomic methods used on sales assistants.
[94]	USA	2015	REBA used in the preparation of laboratory samples.

**Table 9:** Activities of households as employers; undifferentiated goods—and services—producing activities of households for own use.

Reference	Country	Year	Objective
[95]	South Korea	2012	REBA in vacuum cleaning work.
[96]	Sweden	2015	REBA in household chores
[97]	Singapore	2018	REBA used to quantify exposure to musculoskeletal hazards associated with drying clothes

**Table 10:** Education.

Reference	Country	Year	Objective
[98]	Malaysia	2012	RULA and REBA on students while performing their schoolwork.

**Table 11:** Construction.

Reference	Country	Year	Objective
[99]	India	2018	Ergonomic methods in the construction sector.
[100]	USA	2011	Ergonomic methods on prefabricated-panel construction workers.
[101]	Canada	2018	3D body modelling to reduce musculoskeletal disorders in construction.
[102]	China	2019	Creation of an ergonomic assessment tool to apply the REBA method in construction.

**Table 12:** Other activities.

Reference	Country	Year	Objective
[103]	USA	2019	NMQ and REBA in aircraft maintenance.
[104]	Turkey	2017	OWAS and REBA in an electrical equipment factory
[105]	Canada	2012	Eight ergonomic assessment methods in various industrial sectors.
[106]	India	2015	REBA applied to women who carry a load of bricks around their necks.
[107]	Spain	2017	Error detection in the real-life practice of ergonomic assessment methods.
[108]	Canada	2014	REBA in African women who endure head loads during pregnancy.
[109]	Brazil	2016	REBA in the collection of molluscs.
[110]	Brazil	2014	Translation of the REBA method into Portuguese.
[111]	India	2015	REBA in bike repair.
[112]	India	2010	Ergonomic assessment methods in three key Jaipur business sectors.
[113]	India	2010	REBA in Jaipur stone carving.
[114]	South Korea	2017	OWAS, RULA and REBA in the ergonomic assessment of armament cleaning.
[115]	Poland	2014	Comparative analysis of musculoskeletal load assessment methods
[116]	USA	2019	Reliability assessment of the REBA method.
[117]	Canada	2013	RULA and REBA in the ergonomic assessment of casual work.
[118]	Iran	2011	REBA in an engine oil company.
[119]	USA	2010	REBA, RULA and NIOSH used on firefighters and medical emergency technicians.
[120]	USA	2015	REBA and RULA used on librarians.

**Table 13:** Number of publications per scientific journal, knowledge categories (Web of Science), impact factor, rank and quartile (2018).

Journal	P *	Impact Factor	Categories	Rank	Quartile
Work-A Journal of Prevention Assessment and Rehabilitation	17	1.009	Public, environmental and occupational health—SSCI	138/164	Q4
Health Promotion Perspectives	1	No impact factor.			
International Journal of Industrial Ergonomics	14	1.571	Ergonomics—SSCI	7/16	Q2
			Engineering, industrial—SCIE	28/46	Q3
International Journal of Injury Control and Safety Promotion	1	0.87	Public, environmental and occupational health—SSCI	146/164	Q4
Safety Science	1	3.619	Engineering, industrial—SCIE	10/46	Q1
			Operations research and management science—SCIE	16/84	Q1
Journal of Clinical and Analytical Medicine	1	No impact factor.			
Human Factors and Ergonomics in Manufacturing and Service Industries	3	1.000	Ergonomics—SSCI	13/16	Q4
			Engineering, manufacturing—SCIE	45/49	Q4
Global Nest Journal	1	0.869	Environmental sciences—SCIE	232/251	Q4
International Journal on Working Conditions	2	No impact factor.			
Journal of the Faculty of Engineering and Architecture of Gazi University	3	0.652	Engineering, multidisciplinary—SCIE	76/88	Q4
Journal of Back and Musculoskeletal Rehabilitation	1	0.814	Orthopedics—SCIE	65/76	Q4
			Rehabilitation—SCIE	60/65	Q4
International Journal of Environmental Research and Public Health	1	2.468	Environmental Sciences—SCIE	112/251	Q2
			Public, environmental and occupational health—SSCI	38/164	Q1
			Public, environmental and occupational health—SCIE	67/186	Q2
Environmental Health and Preventive Medicine	1	1.568	Public, environmental and occupational health—SSCI	88/164	Q3
			Public, environmental and occupational health—SCIE	120/186	Q3
Asia-Pacific Journal of Public Health	1	1.743	Public, environmental and occupational health—SSCI	73/164	Q2
			Public, environmental and occupational health—SCIE	108/186	Q3
International Journal of Occupational Safety and Ergonomics	3	1.377	Ergonomics—SSCI	9/16	Q3
			Public, environmental and occupational health—SSCI	110/164	Q3
Journal of Occupational Health	1	1.8	Public, environmental and occupational health—SCIE	105/186	Q3
Cahiers Agricultures	1	0.78	Agriculture, multidisciplinary—SCIE	38/57	Q3
			Agronomy—SCIE	63/89	Q3
Iranian Journal of Public Health	1	1.225	Public, environmental and occupational health—SSCI	122/164	Q3
			Public, environmental and occupational health—SCIE	149/186	Q4
Journal of Health and Safety at Work	1	No impact factor.			
Human Factors	1	2.649	Behavioral sciences—SCIE	20/53	Q2
			Engineering, industrial—SCIE	18/46	Q2
			Ergonomics—SSCI	2/16	Q1
			Psychology—SCIE	25/77	Q2
			Psychology, applied—SSCI	24/82	Q2
Dyna Colombia	1	No impact factor.			
Anaesthesia	1	5.879	Anesthesiology—SCIE	4/31	Q1
Aquacultural Engineering	1	2.143	Agricultural, engineering—SCIE	5/13	Q2
			Fisheries—SCIE	17/52	Q2
Journal of Minimally Invasive Gynecology	1	2.547	Obstetrics and Gynecology- SCIE	25/83	Q2
International Journal of Clothing Science and Technology	1	0.752	Materials science, textiles—SCIE	12/24	Q2
Applied Ergonomics	3	2.610	Ergonomics—SSCI	3/16	
			Psychology, applied—SSCI	25/82	
			Engineering, industrial—SCIE	20/46	
Medycyna Pracy	1	0.778	Public, environmental and occupational health—SCIE	171/186	Q4
International Journal of Occupational and Environmental Health	2	0.973	Public, environmental and occupational health—SSCI	141/164	Q4
			Public, environmental and occupational health—SCIE	165/186	Q4
International Journal of Dental Hygiene	1	1.233	Dentistry, oral surgery and medicine—SCIE	68/91	Q3
Ergonomics	1	2.181	Engineering, industrial—SCIE	21/46	Q2
			Ergonomics—SSCI	5/16	Q2
			Psychology—SCIE	38/77	Q2
			Psychology, applied—SSCI	35/82	Q2
Journal of Physical Therapy Science	2	0.392	Rehabilitation—SCIE	61/64	Q4
Brazilian Journal of Physical Therapy	1	1.879	Orthopedics—SCIE	38/76	Q2
			Rehabilitation—SCIE	27/65	Q2
Seefor-South-East European Forestry	1	No impact factor.			
Journal of Construction Engineering and Management	2	2.734	Construction and Building Technology—SCIE	15/63	Q1
			Engineering, civil -SCIE	32/132	Q1
			Engineering, industrial -SCIE	17/46	Q2
International Journal of Precision Engineering and Manufacturing	1	1.779	Engineering, manufacturing	33/49	Q3
			Engineering, mechanical—SCIE	64/129	Q2
Journal of Clinical and Diagnostic Research	1	No impact factor.			
Journal of Chemical Health and Safety	1	No impact factor.			
Design Journal	1	No impact factor.			
Indian Journal of Occupational and Environmental Medicine	1	No impact factor.			
Biosystems Engineering	1	2.983	Agricultural engineering -SCIE	4/13	Q2
			Agriculture, multidisciplinary -SCIE	7/57	Q1
International Journal of Workplace Health Management	1	No impact factor.			
Revista Arvore	1	0.367	Forestry—SCIE	64/67	Q4
Progress in Community Health Partnerships-Research Education and Action	1	0.64	Public, environmental and occupational health—SSCI	153/164	Q4
International Journal of Occupational and Environmental Medicine	1	No impact factor.			
Fresenius Environmental Bulletin	1	0.691	Environmental Sciences—SCIE	240/251	Q4
Laryngoscope	1	2.343	Medicine, research and experimental- SCIE	78/136	Q3
			Otorhinolaryngology—SCIE	12/42	Q2
Logforum	1	No impact factor.			
Journal of Agricultural Engineering	1	No impact factor.			
Health Scope	1	No impact factor.			
Journal of Research in Health Sciences	1	No impact factor.			

* Mode; P = Publications number.
